# Evidence for Cooperative Selection of Axons for Myelination by Adjacent Oligodendrocytes in the Optic Nerve

**DOI:** 10.1371/journal.pone.0165673

**Published:** 2016-11-09

**Authors:** Darragh M. Walsh, Tobias D. Merson, Kerry A. Landman, Barry D. Hughes

**Affiliations:** 1 School of Mathematics and Statistics, University of Melbourne, Victoria, 3010, Australia; 2 Florey Institute of Neuroscience and Mental Health, Parkville, Victoria, 3010, Australia; Yale University School of Medicine, UNITED STATES

## Abstract

The cellular mechanisms that regulate the topographic arrangement of myelin internodes along axons remain largely uncharacterized. Recent clonal analysis of oligodendrocyte morphologies in the mouse optic nerve revealed that adjacent oligodendrocytes frequently formed adjacent internodes on one or more axons in common, whereas oligodendrocytes in the optic nerve were never observed to myelinate the same axon more than once. By modelling the process of axonal selection at the single cell level, we demonstrate that internode length and primary process length constrain the capacity of oligodendrocytes to myelinate the same axon more than once. On the other hand, probabilistic analysis reveals that the observed juxtaposition of myelin internodes among common sets of axons by adjacent oligodendrocytes is highly unlikely to occur by chance. Our analysis may reveal a hitherto unknown level of communication between adjacent oligodendrocytes in the selection of axons for myelination. Together, our analyses provide novel insights into the mechanisms that define the spatial organization of myelin internodes within white matter at the single cell level.

## Introduction

Oligodendrocytes (OLs) are responsible for myelinating the axons of subsets of neurons in the central nervous system. Each OL produces multiple myelin internodes which ensheath numerous axons in their vicinity, insulating them and hence allowing for faster conduction of action potentials. The underlying mechanisms that regulate which axons an OL selects for myelination are starting to be uncovered. Recent studies have identified a role for neuronal activity in defining the set of axons to be myelinated [1–6]. However, it is unknown whether local oligodendrocyte progenitor cells (OPCs) or pre-myelinating OLs interpret axon-derived pro-myelinating cues in a cell autonomous or cooperative manner to effect the myelination of proximal axons.

To investigate this question, we examined two sets of quantitative data published in 2015 by Dumas et al. [7], who analyzed the topographic organization of myelin internodes from clonally labeled OLs in the postnatal mouse optic nerve, a white matter tract in which almost the entire length of every axon is myelinated [8–10]. The morphology of individual OLs was visualized by inducing the expression of different combinations of fluorescent reporter proteins in OLs in a stochastic manner that relied upon low dose administration of tamoxifen to *PLP*:*CreER*^*T2*^*;CAGbow* transgenic mice. Firstly, examination of the concordance between the myelin internodes produced by each OL and the identity of the axons that each OL myelinated revealed no instance in which an OL myelinated a single axon more than once. (We will refer to this finding as ‘Observation A’).

Secondly, Dumas and her colleagues [7] found that adjacent OLs were often observed to form juxtaposed myelin internodes on the same axon i.e. share a common set of axons (we will refer to this finding as ‘Observation B’). This invites the question: do adjacent OLs coordinate their selection of axons for myelination? We investigate the likelihood of each of these sets of observations by reformulating them in terms of classic problems in probability theory. Collectively, our analyses provide new insights into processes operating at the single-cell level that influence the mechanisms by which OLs select axons for myelination within white matter.

## Materials and Methods

We calculate the probabilities that single or adjacent OLs select unique or overlapping populations of axons for myelination. We used the mouse optic nerve as a model white matter tract. To perform our analyses, we first needed to determine the theoretical number of axons that an OL can reach, *N*_*A*_. Analysis of photomicrographs published by Dumas et al. [7] reveals that the maximum length of the primary process of an OL in a mouse optic nerve is ~30 μm, which we take as the radius of influence of an OL. Given that axonal density in the mouse optic nerve is approximately one axon per μm^2^ [11–13], we conclude that each primary process of a single OL could theoretically reach *N*_*A*_ = 2800 axons.

We first analyzed the likelihood of Observation A under the null hypothesis that axon selection for myelination is random. Our calculations relied upon reformulation of the classic birthday problem in probability theory [14]. This problem teaches us that an event that intuitively appears to be highly unlikely, can prove to be more likely than we would anticipate. The classic birthday problem can be summarised as follows. Suppose we choose a random sample of *n* people. Supposing every year contains exactly 365 days and that births are uniformly distributed among those dates, how large does *n* have to be to achieve a probability *p*_*n*_ of at least 0.5 that two or more people share the same birthday (ignoring year of birth)? The surprising answer is that we only require *n =* 23 people, because

$$p_{n}=P(\text{at least two people in a sample share the same birthday})=1-P(\text{no one in a sample shares a birthday})=1-\frac{365\times 364\times \ldots \times (365-n+1)}{365^{n}}$$

and for *n =* 23, *p*_*n*_ = 0.5073.

To apply this methodology to OLs selecting axons, we simply note that *N*_*A*_ = 2800 takes the place of the number of days in a year and that the number of internodes formed by an individual OL takes the place of the sample size *n* in the birthday problem. Dumas et al. [7] performed the three-dimensional reconstruction of 55 OLs in the mouse optic nerve, identifying no instances in which an OL myelinated the same axon more than once. Thus we repeated our calculations 55 times (results are displayed in Table A in S1 Text).

Our analysis of the likelihood of Observation B relied upon reformulation of the ‘coincidence problem’, another classic problem in probability theory with a counter-intuitive solution. Tijms [15] describes the ‘coincidence problem’ as follows. Suppose in a city of one million inhabitants, two people are chosen at random who do not know each other. Suppose also that each person has 500 acquaintances. What is the probability that these two individuals have at least one acquaintance in common? Naively, we might expect this probability to be very low.

This question is an illustration of the hypergeometric distribution, applicable where sampling is done without replacement. The probability of exactly *N*_*s*_ = *X* shared acquaintances is given by the hypergeometric probability distribution function [14,15]

$$P(N_{s}=\text{X shared acquaintances})=\frac{\left(\begin{array}{c} 500\\ X \end{array})(\begin{array}{c} 999,998-500\\ 500-X \end{array}\right)}{\left(\begin{array}{c} 999,998\\ 500 \end{array}\right)},$$

for *X* = 0,1,2,…, 500. For *X* = 0, we find the probability of the two people having no common acquaintances is 0.7787. Thus the probability of them having at least one acquaintance in common is 0.2213. Just as in the birthday problem, our intuition fails us.

We applied this method to analyze the probability of two adjacent OLs sharing *N*_*s*_ axons. In our context, modeling OLs sharing axons by sampling without replacement using the hypergeometric distribution is akin to assuming that an individual OL never myelinates the same axon more than once. The probabilities that we obtained provide an upper bound for the corresponding probabilities when an individual OL repeatedly myelinates an axon, since the number of different axons chosen by each OL may be less than in the unique myelination scenario (see Table A in S2 Text)

## Results

### Analysis of Observation A

In the study of Dumas et al. [7], the topographic organization of myelin internodes from clonally labeled OLs in the postnatal mouse optic nerve was determined for 55 individual OLs. They found that none of these 55 OLs ever myelinated the same axon more than once. Baumann and Pham-Dinh [16] also noted this feature. Given that the density of axons in the optic nerve is approximately 1 axon per μm^2^ [11–13], it might not seem unusual that a single OL would not myelinate an axon more than once if the selection of axons is a random (passive) process. We investigate this intuition below.

In Table A in S1 Text we calculate the probability of unique myelination by each individual OL (*n* = 55) and then multiply these probabilities together to determine the overall probability of never observing unique myelination. For illustration we analyze two specific experimental findings from Dumas et al. [7]. The first concerns an OL from a mouse at postnatal day 10 (P10) which produced the least number of internodes, four, of all the mice examined by Dumas et al. [7]. Our second test concerns an OL from an adult mouse that produced the maximum number of internodes, 59, of all the mice examined.

What is the probability that an OL, which selects four axons to myelinate from a sample of 2800 axons, will not choose the same axon more than once? Analogous to a calculation done for the birthday problem, this probability is

$$\frac{2800\times 2799\times 2798\times 2797}{2800^{4}}≅0.9979.$$

That is, the probability that an OL will myelinate a unique set of axons is 0.9979 if the OL produces just 4 internodes (as determined at P10 in the mouse optic nerve). Similarly, the probability of observing unique myelination when the OL produces 59 internodes (the maximum number of internodes observed for an OL in the adult optic nerve) is approximately 0.5404. Each observation is assumed independent so the results from each experiment (15 OLs at P10, 15 OLs at P22, 13 OLs at P45 and 12 adult OLs) may be multiplied to assess the likelihood of observing these results (the result of each calculation is displayed in Table A in S1 Text). This yields

P(single OL never myelinates an individual axon more than once)≅0.1015

Note that Dumas and her colleagues [7] only quantified the number of internodes extended for 12 of the 26 OLs examined in the adult mouse optic nerve. However, they still noted that none of the remaining 14 OLs ever myelinated the same axon more than once. Given that OLs in the adult optic nerve extend many more internodes on average than those in development, the probability of observing unique myelination is considerably smaller.

**Fig 1:**
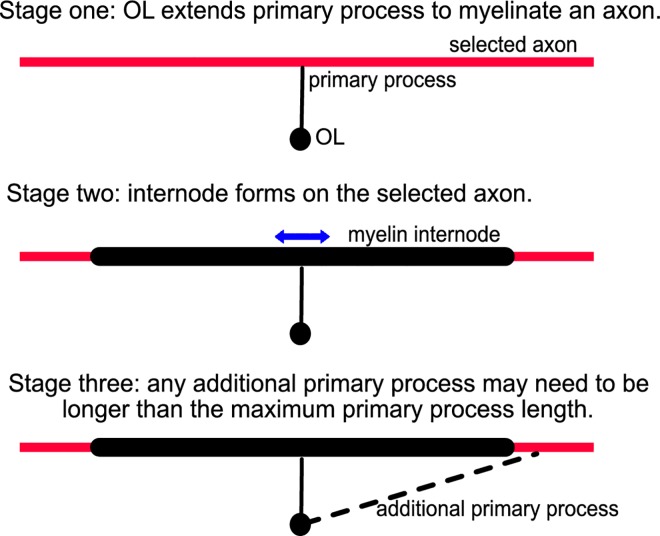
Schematic depicting the physical constraints limiting repeated myelination of an axon by a single OL. Given the average myelin internode length (approximately 130 μm) compared to the maximal length of an OL primary process (30 μm), the probability of repeated myelination of an individual axon by a single OL is reduced. The scenario depicted is where the angle of incidence between the primary process and the axon is 90 degrees and the point of contact lies at the midpoint of the internode. In the simulation which quantifies this reduction in the probability of repeated myelination both the angle of incidence and the location of the point of contact are chosen randomly (S1 Fig).

However, the elaboration of each internode (on average approximately 130 μm in length [7]) may exclude these myelinated axonal segments from repeated myelination. Thus if an OL were to myelinate a given axon more than once, any additional primary processes may need to be longer than the maximum primary process length (Fig 1). We developed a simulation model where an individual OL’s choice of axons to myelinate is subject to this constraint arising from the nonzero internode length and finite maximum primary process length.

Imposing this constraint increased the probability of observing individual OLs myelinating a unique set of axons in 55 trials from 0.1015 to 0.3156. (Table A in S1 Text). This probability of 0.3156 should be interpreted as a lower bound since all axons were assumed to be *a priori* unmyelinated. In reality, since myelination does not occur instantaneously and hence the process of axonal selection occurs incrementally, one would predict that ever increasing competition between OLs for internode placement along axons progressively restricts access to unmyelinated axonal segments. In other words, the likelihood of observing any instance of repeated myelination with a sample size of 55 OLs is predicted to be at most ~68%. Among the 55 OLs examined in Dumas et al. (2015), no OL was observed to myelinate the same axon more than once. We conclude that there is insufficient evidence to support the hypothesis that OLs actively avoid myelinating the same axon more than once and propose that the physical constraints of internode and primary process length play an important role in preventing instances of repeated myelination of the same axon.

### Analysis of Observation B

We next analyze the likelihood of Observation B from Dumas et al. [7], namely that adjacent OLs frequently myelinated a common set of axons. To investigate this, we assume based on the experimental observations [7] that myelin internodes elaborated by a single OL myelinate a unique sets of axons (Observation A).

The number of shared axons *N*_*S*_ has the probability distribution

$$P(N_{S}=X)=\frac{\left(\begin{array}{c} N_{I}\\ X \end{array})(\begin{array}{c} N_{A}-N_{I}\\ N_{I}-X \end{array}\right)}{\left(\begin{array}{c} N_{A}\\ N_{I} \end{array}\right)},$$

where *N*_*I*_ is the number of internodes produced by an OL and the expected number of shared axons is *E*(*N*_*S*_) = *N*_*I*_^2^/*N*_*A*_.

**Fig 2:**
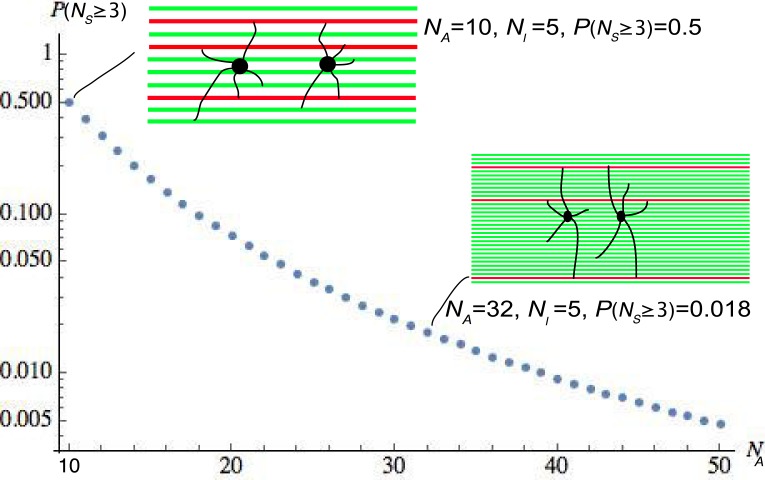
The probability of two adjacent OLs myelinating at least three axons in common, P(*N*_*S*_ ≥ 3), with the number of axons within reach (*N*_*A*_) varied. The number of internodes formed *N*_*I*_ by each OL is chosen to be five for illustrative purposes. Green horizontal lines in the insets denote axons and red horizontal lines the shared axons. The black filled circles represent OL cell bodies from which processes extend. A logarithmic scale is used on the vertical axis. Perhaps unexpectedly, with *N*_*A*_ = 10 the probability of observing at least three shared axons is 0.5 and not a much lower probability. Note that we have used *N*_*A*_ = 2800 in our calculations in the text, which corresponds to a density of one axon per μm^2^ [11–13] and an approximate maximum primary process length of 30 μm [6].

Dumas and colleagues [7] observed several examples where two adjacent OLs shared at least three axons in common. To obtain a conservative estimate of the probability of this occurrence, we used the observed maximum number of internodes for any OL up to postnatal day 45, *N*_*I*_ = 18. For illustrative purposes, Fig 2 displays the dependence of the probability of two adjacent OLs sharing at least 3 axons, *P*(*N*_*S*_ ≥ 3), on the number of axons *N*_*A*_ with *N*_*I*_ = 5. As already noted, the actual value of *N*_*A*_ is approximately 2800.

With *N*_*A*_ = 2800 and *N*_*I*_ = 18, reflecting the maximum number of internodes observed at P45, the expected number of shared axons is *E*(*N*_*S*_) = 0.116. The probability of no axons being shared by two OLs is *P*(*N*_*S*_ = 0) = 0.890, so the probability of at least one axon being shared is 0.110. The probability of at least two shared or at least three shared axons is 0.0056 and 1.72 × 10^−4^ respectively. Thus, if we assume that axonal selection by adjacent OLs is random, the expected probability of observing two or more of the same axons being myelinated by two adjacent OLs is exceedingly low. S1 Table reveals that this conclusion is not sensitive to the value of *N*_*A*_. For example, if the maximum primary process length is 40μm rather than 30μm then the value of *N*_*A*_ is approximately 5000 (still assuming a density of 1 axon per μm^2^). From S1 Table we see that the probability of observing at least three shared axons by adjacent OLs is reduced to approximately 2.92× 10^−5^. Removing the assumption that each individual OL myelinates a unique sets of axons makes only a slight numerical change to our results and no change to our conclusions (Table A in S1 Text).

In contrast to the low probabilities of shared myelination among two adjacent OLs that we predict, Dumas and colleagues [7] provide examples of at least three shared axons from an optic nerve at P20 and at least two shared axons from an adult optic nerve and note that adjacent OLs shared axons in all optic nerves analyzed. These empirical observations are inconsistent with probabilistic analysis, thus we conclude that the selection of axons by adjacent OLs is an active regulated process.

## Discussion

In this study we determined the probabilities that single or adjacent OLs in the mouse optic nerve select a unique or overlapping population of axons for myelination based on the assumption that the process of axonal selection is random. We compared our predictions to empirical observations of axonal selection by clonally labelled oligodendrocytes in the mouse optic nerve described by Dumas et al. [7]. Using probabilistic analyses, we investigated two key observations described by Dumas and her colleagues: 1) that individual OLs were never observed to myelinate the same axon more than once; 2) that adjacent OLs were frequently observed to myelinate a shared population of two or three axons. In respect of the first observation, our results demonstrate that the observed frequency of unique myelination by OLs is insufficient to exclude the hypothesis that axonal selection is random. Dumas and her colleagues proposed that a process of active self-avoidance or self-repulsion could prevent the formation of adjacent internodes arising from the same OL during myelination [7]. Our analysis provides an alternate explanation for these observations. Our simulation model of axonal selection by OLs reveals that the disparity between an OL’s primary process length and the length of the myelin internodes that they elaborate imposes significant physical constraints that deter repeated myelination of the same axon. Although we cannot exclude the possibility that a process of self-repulsion also contributes to the observation of unique myelination, we conclude that self-repulsion of OL processes is not a necessary prerequisite for the selection of unique axons.

In respect of the second observation noted by Dumas and her colleagues [7], that a subset of myelin internodes produced by adjacent OLs were often juxtaposed along two or three of the same axons, we conclude that this phenomenon has negligible probability of occurring by chance. It may be argued that the sharing of axons by adjacent OLs is the result of the OLs being independently but simultaneously controlled by fine-tuned environmental stimuli without the need for cooperation amongst adjacent OLs. This interpretation is more suitable for white matter tracts that are partially myelinated. There would need to be evidence for variability in localised pro-myelinating cues along the length of the axon that results in local zones of myelination. In order to establish the observed pattern of multiple shared adjacent internodes, these active pro-myelinating zones would have to spatially and temporally coincide since the timeframe for OL differentiation and myelination is short [17]. The data obtained by Dumas et al. [7] relate to the optic nerve where each nerve is (almost) completely myelinated. If electrical activity drives myelination in the optic nerve then the entire length of the axon should be myelinated within a similar timeframe. The more parsimonious explanation is that adjacent OLs are guided by one another as to which axons they target for myelination.

Our analysis supports the notion that the sharing of axons by adjacent OLs is a coordinated active process. We infer that this level of coordination may reflect a generic process of communication between neighboring OLs that enables the process of axonal selection to be tightly coordinated. We propose that coordinated regulation among neighboring OLs in the selection of axons for myelination provides a mechanistic link between activity-dependent processes that promote myelination and the generation of precise topographic patterns of myelin that are likely necessary for synchronizing neuronal conduction among populations of functionally related axons. A key objective for future research will be to explore potential mechanisms by which adjacent OLs could communicate with one another to coordinate axonal selection.

## Supporting Information

S1 Fig Schematic of how an OL in our simulation model may myelinate the same axon twice given the internode and maximum primary process length constraints. (PDF)

S1 Table Sensitivity analysis. (PDF)

S1 Text Calculating the overall probability of observing unique myelination given the data in Dumas et al. (2015). (PDF)

S2 Text Generalizations. (PDF)

S3 Text Triply shared myelination. (PDF)
