# Supplementary material for: Evidence for Cooperative Selection of Axons for Myelination by Adjacent Oligodendrocytes in the Optic Nerve
Source: PLoS One. 2016 Nov 9;11(11):e0165673. doi: 10.1371/journal.pone.0165673 (PMC5102443; doi:10.1371/journal.pone.0165673)
Supplement: S1 Fig — (PDF) [file pone.0165673.s001.pdf]

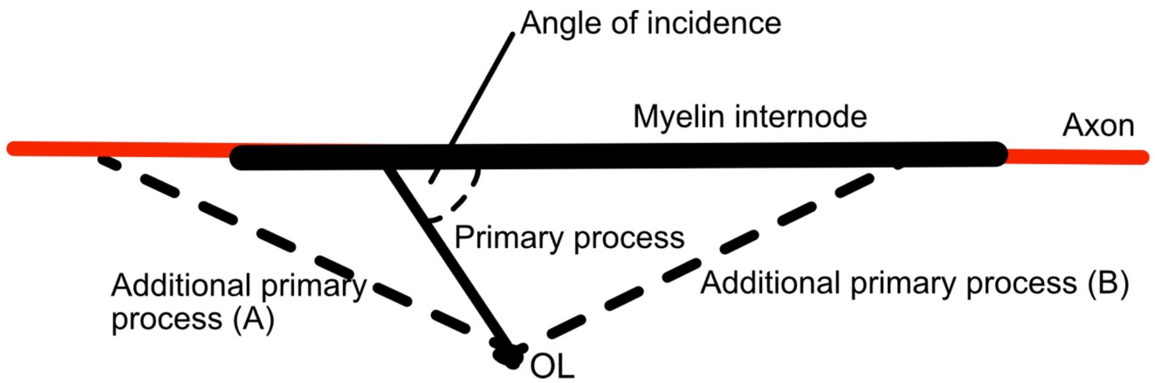


**S1 Fig. Schematic of how an OL in our simulation model may myelinate the same axon twice given the internode and maximum primary process length constraint. The primary process length, angle of incidence, myelin internode length and proportion of the internode to the right of the primary process intersection were chosen randomly from biologically relevant ranges. All primary processes have maximum length of 30 µm**. In this representation, an additional primary process (B) would be excluded since it is not long enough to reach a bare axon segment whereas an additional primary process (A) is viable as it can reach a bare axon segment.
