# Supplementary material for: Evidence for Cooperative Selection of Axons for Myelination by Adjacent Oligodendrocytes in the Optic Nerve
Source: PLoS One. 2016 Nov 9;11(11):e0165673. doi: 10.1371/journal.pone.0165673 (PMC5102443; doi:10.1371/journal.pone.0165673)
Supplement: S1 Table — (PDF) [file pone.0165673.s002.pdf]

S1 Table. Sensitivity analysis

N_A	Probability of observing unique myelination of axons by 55 individual OLs (without constraint)	Probability of two adjacent OLs sharing at least three axons when $N_I = 18$
600	1.97×10^{-5}	0.01397
900	0.0008	0.00455
1200	0.0047	0.00201
1500	0.0138	0.00106
1800	0.0283	0.00062
2100	0.0472	0.00040
2400	0.0692	0.00027
2700	0.0932	0.00019
3000	0.1183	0.00014
3300	0.1437	0.00011
3600	0.1690	8.18×10^{-5}
3900	0.1939	6.45×10^{-5}
4200	0.2181	5.18×10^{-5}
4500	0.2414	4.23×10^{-5}
4800	0.2639	3.49×10^{-5}
5100	0.2855	2.92×10^{-5}
5400	0.3061	2.46×10^{-5}
5700	0.3258	2.10×10^{-5}
6000	0.3446	1.80×10^{-5}

S1 Table. Dependence of our calculations upon the number of axons within reach of each OL. Decreasing N_A from its base value of 2800 discussed in the text (which corresponds to an axonal density of 1 axon per μm^2 and a maximum primary process length of 30 μm) decreases the probability of observing unique myelination of axons by individual OLs (ignoring the constraint) but increases the probability of OLs sharing axons. Note that if we the density of axons remains at 1 axon per μm^2 and we increase the maximum primary process length to 40 μm, then N_A is approximately 5100. Our conclusion that OLs sharing axons is an active process is clearly independent of the number of axons each OL can reach, the only parameter in this study.
