# Supplementary material for: Evidence for Cooperative Selection of Axons for Myelination by Adjacent Oligodendrocytes in the Optic Nerve
Source: PLoS One. 2016 Nov 9;11(11):e0165673. doi: 10.1371/journal.pone.0165673 (PMC5102443; doi:10.1371/journal.pone.0165673)
Supplement: S1 Text — (PDF) [file pone.0165673.s003.pdf]

S1 Text. Calculating the overall probability of observing unique myelination given the data in Dumas et al. [7]

Let us first ignore the constraint imposed by the internode and maximum primary process length on repeated myelination of the same axon by an individual OL. If an OL were to choose N_I axons randomly, possibly choosing each axon more than once, from a pool of N_A axons that it can reach, the probability of it making only unique choices is

$$P(\text{OL chooses unique axons to myelinate}) = \binom{N_A}{N_I} \frac{N_I!}{{N_A}^{N_I}}. \quad \text{(S1)}$$

To determine the probability of never observing repeated myelination of an axon by an individual OL we need to know the number of internodes produced by each of the 55 OLs that were analyzed. These can easily be derived from Fig 7 I and Fig 11 I in Dumas et al. (2015) [7]. Performing the calculation using the formula above for each of these OLs, we find that the probability of unique myelination is approximately 0.1015. This calculation ignores the 14 adult OLs for which the number of processes was not counted though the absence of repeated myelination of an axon was noted. Hence the true probability is much lower.

However, any secondary process seeking to myelinate an axon already chosen by that OL is subject to a constraint since the maximum process length may not be long enough to reach a viable location on the axon not covered by myelin. This is explained graphically in Fig 1 in the main text.

To quantify the likelihood of observing only unique myelination of axons by individual OLs subject to the additional internode length constraint we developed a simulation model where the primary process length (0-30 μm), angle of incidence, internode length (mean values for each OL as displayed in Dumas et al. [7]) and proportion of the internode length that lies to the right of the axon-primary process intersection point were all chosen randomly within biological observed ranges. The value of the internode length was selected at random from the mean values for each OL displayed in Dumas et al. [7] Fig 11 H and Fig 7 H. The additional primary process was assumed to choose viable locations for ensheathing the axon over areas already myelinated. This model is displayed graphically in S1 Fig. The probability of observing 55 OLs each myelinating unique axons is raised to 0.3156 when the internode and maximum primary process length constraint is enforced.

P10 OLs			P22 OLs			P45 OLs			Adult OLs		
N_I	P exact	P sim	N_I	P exact	P sim	N_I	P	P sim	N_I	P exact	P sim
11	0.9805	0.9899	5	0.9964	0.9984	16	0.9580	0.9844	9	0.9872	0.9932
8	0.9900	0.9949	8	0.9900	0.9959	17	0.9525	0.9817	12	0.9767	0.9872
12	0.9767	0.9882	8	0.9900	0.9961	18	0.9467	0.9794	7	0.9925	0.9956
9	0.9872	0.9933	9	0.9872	0.9944	16	0.9580	0.9837	6	0.9947	0.9971
6	0.9947	0.9970	10	0.9840	0.9934	10	0.9840	0.9938	27	0.8818	0.9338
6	0.9947	0.9972	7	0.9925	0.9971	8	0.9900	0.9961	25	0.8981	0.9450
7	0.9925	0.9962	6	0.9947	0.9978	7	0.9925	0.9973	18	0.9467	0.9705
12	0.9767	0.9881	9	0.9872	0.9947	5	0.9964	0.9987	11	0.9805	0.9891
11	0.9805	0.9897	9	0.9872	0.9945	7	0.9925	0.9972	38	0.7771	0.8721
12	0.9767	0.9881	9	0.9872	0.9946	8	0.9900	0.9963	59	0.5404	0.7177
10	0.9840	0.9919	15	0.9631	0.9845	10	0.9840	0.9938	42	0.7342	0.8448
9	0.9872	0.9934	15	0.9631	0.9842	11	0.9805	0.9928	7	0.9925	0.9959
8	0.9900	0.9954	17	0.9525	0.9793	10	0.9840	0.9940			
6	0.9947	0.9971	11	0.9805	0.9919						
4	0.9979	0.9989	6	0.9947	0.9977						

Table A. Data on the number of internodes formed by each OL derived from the graphics in Fig 7 I and Fig 11 I in Dumas et al. [7] together with the probabilities *P* exact and *P* sim that each OL will not myelinate any axon more than once (where $N_A = 2800$). *P* exact is derived from equation (S1) and ignores the internode and maximum primary process length constraint. *P* sim is derived from our simulation model, which implements the constraint. The product of all 55 *P* exact probabilities gives approximately 0.1015 whilst the product of all 55 *P* sim probabilities is naturally higher and equals 0.3156. If instead we assume that the point of contact between the primary process and the internode occurs at the midpoint of the internode (as observed in a zebrafish study [18]), then all the P sim entries above change. Multiplying all these new P sim values together results in the probability of never observing repeated myelination of an axon by an individual OL increasing from 0.3156 to 0.6295.

Additional Reference

[18] Snaidero N, Mobius W, Czopka T, Hekking LHP, Mathisen C, Verkeij D, et al. Myelin Membrane Wrapping of CNS Axons by PI(3,4,5) P3–Dependent Polarized Growth at the Inner Tongue. Cell 2014 **156**: 277–290.
