# Supplementary material for: Evidence for Cooperative Selection of Axons for Myelination by Adjacent Oligodendrocytes in the Optic Nerve
Source: PLoS One. 2016 Nov 9;11(11):e0165673. doi: 10.1371/journal.pone.0165673 (PMC5102443; doi:10.1371/journal.pone.0165673)
Supplement: S2 Text — (PDF) [file pone.0165673.s004.pdf]

S2 Text. Generalizations

a) The role of spatial bias in axon selection

We assumed that all axons were equally likely to be chosen for myelination. If an OL were to favour nearby axons for myelination over axons further away, then the probability of no repeated myelination of an axon would be less than the value of 0.1015 calculated in Table A in S1 Text. This is because more axons would be chosen from a smaller sub-sample (i.e. the nearby axons) of all the axons it can reach. So the probability of never observing repeated myelination (ignoring the internode length constraint) would be even smaller than 0.1015, hence our estimate is conservative.

We now investigate the role of spatial effects in assigning probabilities to OLs sharing multiple axons. If nearby axons are favored for myelination then the likelihood of shared axons increases because selections are being made from a smaller sample of axons. We can estimate these probabilities using the law of large numbers [14, 15] where the relative probability of selection falls linearly from one (nearest axon, axon 1 say) to close to zero (furthest axon, axon 2800 say). We find that there is a slight increase in the probability of shared axons when nearby axons are favored, Table A below, but our conclusion that axon selection by adjacent OLs is an active coordinated process remains.

b) Non-unique myelination:

We interpreted our analysis of the experiments of Dumas et al. [7] as evidence that individual OLs avoid myelinating an axon more than once. We used this conclusion by basing our analysis of OLs sharing axons on the hypergeometric distribution, which is suitable for modeling a process as sampling without replacement. We refer to this assumption as unique myelination. However, had we chosen to ignore our conclusion and start afresh we would base our analysis on a model of sampling with replacement. We refer to this assumption as non-unique myelination.

The unique myelination scenarios provide upper bounds for the probabilities since there are a greater number of different axon selections in this case and hence a larger probability of an overlap between the axon choices of the two OLs, as observed in Table A below.

Note that in Table A below, and in the main text, we sought a conservative estimate of the probability of shared myelination by choosing $N_I = 18$. This was the maximum number of internodes formed by an OL throughout the whole of the development period (up to P45). If instead we had chosen to use the mean number of internodes observed for OLs at P22, $N_I = 10$, the probability of no shared axons is 0.965, the probability of at least one shared is 0.035, the probability of at least two or at least three axons shared is 5.09×10^{-4} and 3.89×10^{-6} respectively. These values should be compared to the "Unique myelination: exact" column in Table A below.

	Unique myelination: exact	Unique myelination with nearby axon bias: simulation	Non-unique myelination: simulation	Non-unique myelination with nearby axon bias: simulation
$P(N_S = 0)$	0.8901	0.8558	0.8902	0.8573
$P(N_S \geq 1)$	0.1099	0.1442	0.1098	0.1427
$P(N_S \geq 2)$	0.0056	0.0098	0.0056	0.0095
$P(N_S \geq 3)$	1.72×10^{-4}	3.81×10^{-4}	1.63×10^{-4}	3.61×10^{-4}

Table A. Probability of two adjacent OLs ($\boldsymbol{N_A = 2800, N_I = 18}$) sharing $\boldsymbol{N_S}$ axons calculated with the assumption that an OL myelinates unique axons (exact hypergeometric formula and a simulation model with nearby axon bias) and without this assumption (simulation model without and with nearby axon bias). The models without any bias assume that all axons have the same probability of being selected. In contrast, the simulation models with bias assume the relative probability that an axon is selected decreases linearly from one for the nearest axon (i.e. if this axon is chosen it will be myelinated) to almost zero for the furthest axon it can reach (i.e. if this axon is selected it is unlikely to be myelinated in which case another axon is chosen). If selection favors nearby axons then the probability of multiple axons being shared increases but our conclusions remain unchanged. Simulations were averaged over one million realizations.
