# Supplementary material for: Evidence for Cooperative Selection of Axons for Myelination by Adjacent Oligodendrocytes in the Optic Nerve
Source: PLoS One. 2016 Nov 9;11(11):e0165673. doi: 10.1371/journal.pone.0165673 (PMC5102443; doi:10.1371/journal.pone.0165673)
Supplement: S3 Text — (PDF) [file pone.0165673.s005.pdf]

S3 Text. Triply shared myelination

Although we have no current experimental data on the occurrence of three adjacent OLs myelinating a set of common axons, the following calculation shows that it has a negligible probability of occurring due to chance alone.

We determine the exact probability that three adjacent OLs myelinate the same axon, assuming each OL has the same population of axons within its radius of influence and that there is no nearby axon selection bias. We again choose $N_A = 2800$ and $N_I = 18$. Consider the second and third OLs. These must share T axons, where T = 0, 1, 2, … N_I. We denote the event that OL1 shares N_S axons with OL2 and OL3 by A_{Ns} and denote the event that OL2 and OL3 share T axons by B_T. We have a partition of the sample space since only one of the T events $\{B_T\}$ must occur. The law of total probability then tells us that the probability of the three OLs sharing N_S axons is

$$P(\text{these 3 OLs share } N_S \text{ axons}) = \sum_{T=0}^{N_I} P(A_{Ns}|B_T)P(B_T),$$

where

$$P(A_{Ns}|B_T) = \frac{\binom{T}{N_S}\binom{2800-T}{18-N_S}}{\binom{2800}{18}} \text{ and } P(B_T) = \frac{\binom{18}{T}\binom{2800-18}{18-T}}{\binom{2800}{18}}.$$

Here $P(A_{Ns}|B_T)$ is the conditional probability of OL1 sharing N_S axons with OL2 and OL3 given that OL 2 and OL3 share T axons.

The probability of at least one shared axon is 7.44×10^{-4}. The probability of at least two shared axons is 2.33×10^{-7} and the probability of at least three shared axons is 4.07×10^{-11}.
